# Supplementary material for: Buckypaper-Based Nanostructured Sensor for Port Wine Analysis
Source: Sensors (Basel). 2022 Dec 12;22(24):9732. doi: 10.3390/s22249732 (PMC9783348; doi:10.3390/s22249732)
Supplement: Supplementary file 1 [file sensors-22-09732-s001.zip › sensors-1952299-supplementary.pdf]

Supplementary Materials

Buckypaper-Based Nanostructured Sensor for Port Wine Analysis

Luiza Ferreira [1], Paula Pinheiro [2], Newton Barbosa Neto [1] and Marcos Reis [1,2,*]

[1] Programa de Pós-Graduação em Ciência e Engenharia de Materiais, Universidade Federal do Pará, Ananindeua 67130-660, PA, Brazil
[2] Programa de Pós-Graduação em Engenharia de Recursos Naturais da Amazônia, Instituto de Tecnologia, Universidade Federal do Pará, Belem 66075-110, PA, Brazil
* Correspondence: marcosallan@ufpa.br

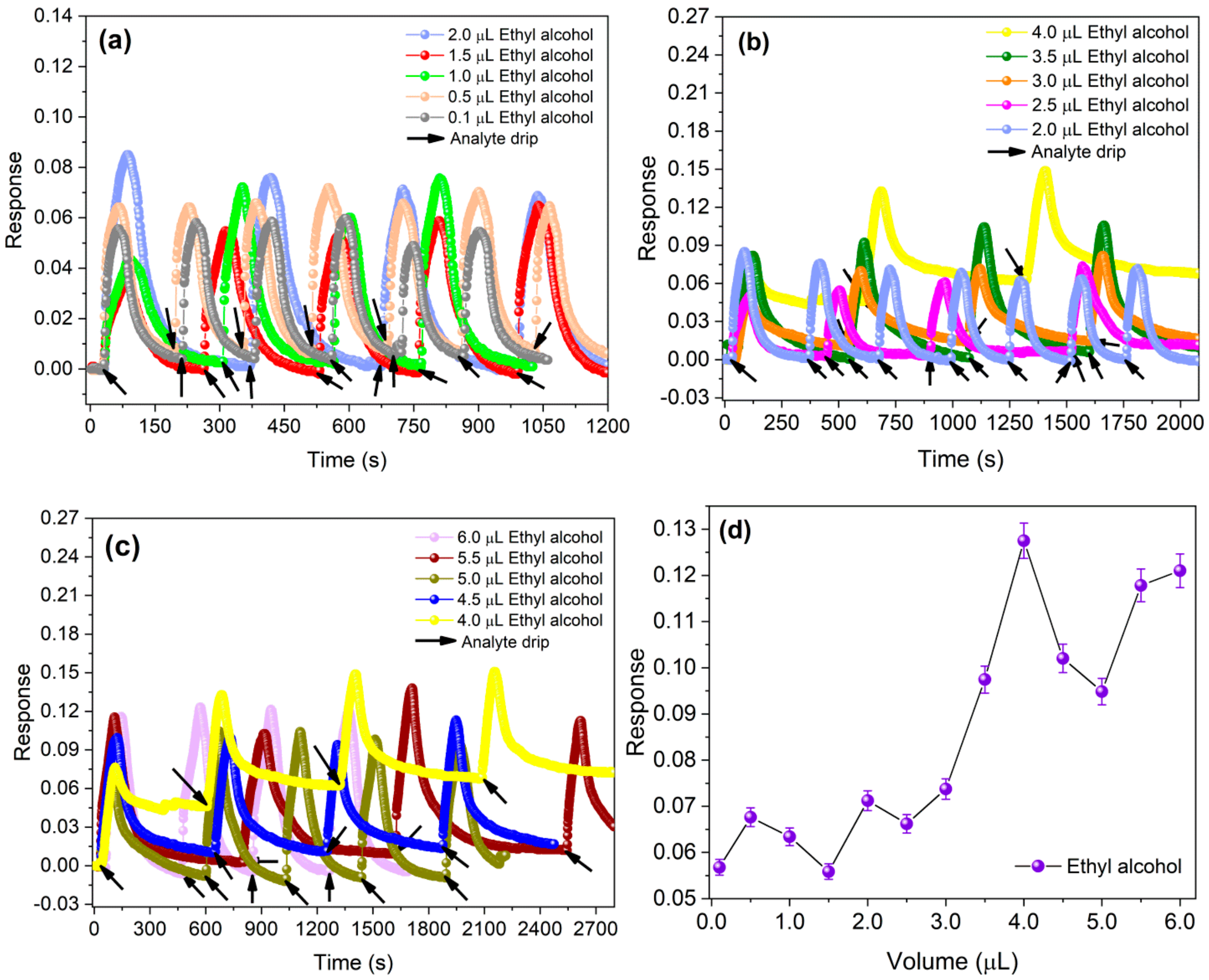

Figure S1. Dynamic response of the sensor in the presence of volumes of ethyl alcohol in (**a**) 0.1 to 2.0 μL, (**b**) 2.0 to 4.0 μL and (**c**) 4.0 to 6.0 μL demonstrating the best performance to 4.0 μL of solvent. Comparative analysis of the response in (**d**) shows 0.12 ± 0.03 for the volume of 4.0 μL of ethyl alcohol.

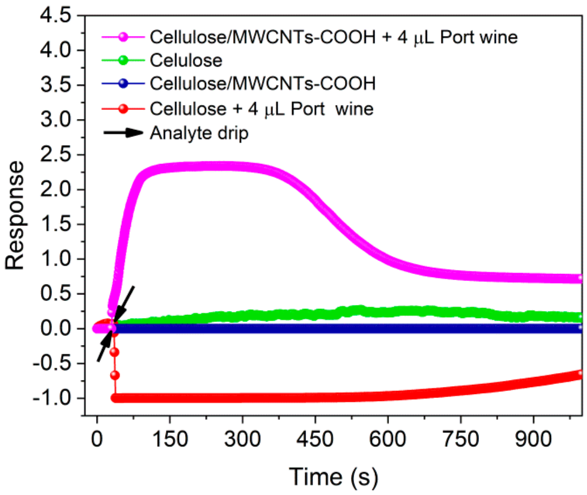

Figure S2. Comparative response curves for samples of cellulose without MWCNTs-COOH (filter paper) and cellulose with MWCNTs-COOH (buckypaper) without and after exposure to 4 μL of unadulterated Port wine.

PCA provides an estimate of sensor distinguishability by projecting points into distinct graph quadrants. However, for a complete PCA analysis, it would be necessary to expose the sensor to more measurements for each analyte, thus generating clusters of points that would allow the visualization of existing patterns of similarity and differentiation.

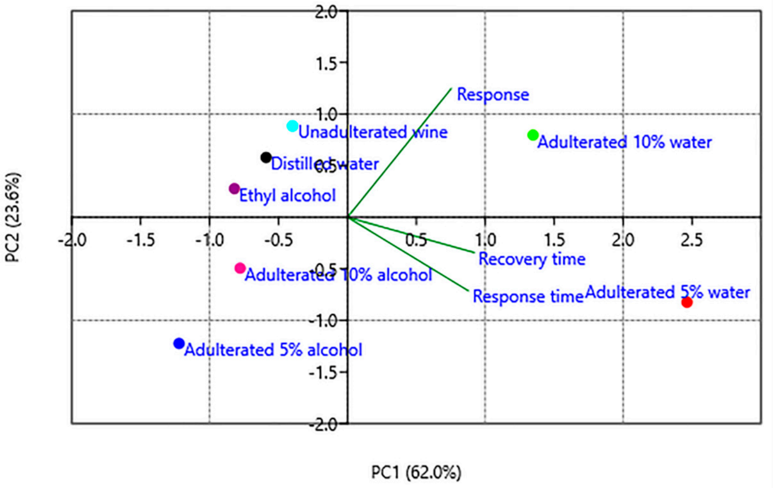

Figure S3. PCA Biplot for 4.0 μL of distilled water, ethyl alcohol, unadulterated and adulterated Port wine with 5 vol.% and 10 vol.% distilled water and ethyl alcohol as a function of the variables response, response time and recovery time showing an indication the distinguishability of the sensor to unadulterated and adulterated Port wine with distilled water and ethyl alcohol.

Table S1. Mean of response and recovery times and their respective standard deviations (SD) for different cycles of the sensor in the presence of individual analytes and adulterated Port wine with 5 vol.% and 10 vol.% of distilled water and ethyl alcohol.

Analyte	Response Time (s)	Standard deviation (SD)	Recovery Time (s)	Standard deviation (SD)
Distilled water	78.0	12.72	181.5	9.19
Ethyl alcohol	65.5	5.74	126.5	12.23
Unadulterated wine	95.5	31.29	163.0	61.11
Adulterated 5% water	2,240	763.67	2,688	876.81
Adulterated 10% water	103.3	70.94	3,035	404.75
Adulterated 5% alcohol	10.0	3.60	1,030.6	116.86
Adulterated 10% alcohol	105.0	95.0	870.5	377.0

Table S2. Data extracted via covariance matrix showing the variance explained by each Principal Component (PC).

PC	Eigenvalue	Variance (%)
1	186.013	62.004
2	0.707915	23.597
3	0.431954	14.398

Table S3. Score data for the samples as a function of the three PCs generated.

Score	PC 1	PC 2	PC 3
Distilled water	-0.58985	0.58009	0.39597
Ethyl alcohol	-0.82017	0.27765	0.35793
Unadulterated wine	-0.39834	0.88516	0.48128
Adulterated 5% water	24.634	-0.82341	0.63001
Adulterated 10% water	13.463	0.79646	-11.803
Adulterated 5% alcohol	-12.221	-12.225	-0.50166
Adulterated 10% alcohol	-0.77917	-0.49344	-0.18325
